# Cytotoxic-Ag-Modified Eggshell Membrane Nanocomposites as Bactericides in Concrete Mortar

**DOI:** 10.3390/ijms242015463

**Published:** 2023-10-23

**Authors:** Samuel Tomi Aina, Hilda Dinah Kyomuhimbo, Barend Du Plessis, Vuyo Mjimba, Nils Haneklaus, Hendrik Gideon Brink

**Affiliations:** 1 Department of Chemical Engineering, University of Pretoria, Pretoria 0002, South Africa; samuel.aina@tuks.co.za (S.T.A.); u21830658@tuks.co.za (H.D.K.); barend.duplessis@up.ac.za (B.D.P.); 2 Human Sciences Research Council, 134 Pretorius Street, Pretoria 0083, South Africa; vmjimba@hsrc.ac.za; 3 Td Lab Sustainable Mineral Resources, University for Continuing Education Krems, Dr.-Karl-Dorrek-Straße 30, 3500 Krems, Austria; nils.haneklaus@donau-uni.ac.at

**Keywords:** concrete, bactericide, silver nanoparticles, eggshell membrane, nanocomposite

## Abstract

Against the backdrop of escalating infrastructure budgets worldwide, a notable portion—up to 45%—is allocated to maintenance endeavors rather than innovative infrastructure development. A substantial fraction of this maintenance commitment involves combatting concrete degradation due to microbial attacks. In response, this study endeavors to propose a remedial strategy employing nano metals and repurposed materials within cement mortar. The methodology entails the adsorption onto eggshell membranes (ESM) of silver nitrate (ESM/AgNO_3_) or silver nanoparticles (ESM/AgNPs) yielding silver–eggshell membrane composites. Subsequently, the resulting silver–eggshell membrane composites were introduced in different proportions to replace cement, resulting in the formulation of ten distinct mortar compositions. A thorough analysis encompassing a range of techniques, such as spectrophotometry, scanning electron microscopy, thermogravimetric analysis, X-ray fluorescence analysis, X-ray diffraction (XRD), and MTT assay, was performed on these composite blends. Additionally, evaluations of both compressive and tensile strengths were carried out. The mortar blends 3, 5, and 6, characterized by 2% ESM/AgNO_3_, 1% ESM/AgNPs, and 2% ESM/AgNPs cement replacement, respectively, exhibited remarkable antimicrobial efficacy, manifesting in substantial reduction in microbial cell viability (up to 50%) of typical waste activated sludge. Concurrently, a marginal reduction of approximately 10% in compressive strength was noted, juxtaposed with an insignificant change in tensile strength. This investigation sheds light on a promising avenue for addressing concrete deterioration while navigating the balance between material performance and structural integrity.

## 1. Introduction

Concrete is undoubtedly the most used construction material having found acceptance in a wide variety of applications. Notable is the fact that this mixture of cement, fine aggregate, coarse aggregate, and water (in the case of concrete) or just cement sand and water (mortar) is nowhere near its end of life [1,2,3]. Statista [4] reported that 4.1 billion tons of cement was produced in 2022 as against 1.39 billion tons in 1995.

As versatile as it is, one principal dilemma of concrete structures is its high cost of maintenance which has been seen to go as high as 45% of the total infrastructure budget in the UK and EU [2]. This expensive maintenance has been necessitated due to environmental factors, human factors, use over time, and microbial attack (especially in wet conditions) [5].

The need to prolong the life of concrete while reducing the cost of maintenance has, therefore, necessitated various research with some aimed at low-weight (foamed) concrete, improving mechanical properties, surface treatment inhibiting bacterial growthand biofilm formation. Among many other antimicrobial agents, silver and silver nanoparticles (AgNPs) are some of the most promising bacterial inhibitors [5,6,7,8,9].

The size, shape, surface charge, concentration, and colloidal state are the key physio-chemical factors that significantly influence the antimicrobial effectiveness of silver nanoparticles (AgNPs). Some of the shapes documented in the literature include oval, spherical, cubic, cylinder, and triangular, which are sometimes attributed to different synthesis techniques [10,11,12]. These synthesis techniques include physical, chemical, or green methods. The chemical method, which involves the use of reducing agents and stabilizers such as formaldehyde, hydrazine, and sodium borohydride, is one such technique [13].

Eggs are characterized as being porous, bioceramic, calcareous, and oval. Chicken eggs possess the necessary strength to withstand physical and pathogenic threats while still facilitating the exchange of water and gases necessary for embryo development [14,15,16]. Lining the walls of the eggshell is the shell membrane (ESM). ESM is a fibrous biomaterial that possesses a high surface area and excellent adhesion ability [17]. As indicated in Li et al. (2017) [18], the adsorption of metal ions by ESM is facilitated by electrostatic, hydrogen bonding, and van der Waals forces, which are activated when ESM is exposed to these ions.

Globally, egg production continues to rise with each passing year and with this increase comes the need to manage the shell waste. In 2008, 62 million metric tons were recorded. This grew to 76.7 million metric tons in 2018 and 86.3 million metric tons in 2021 [19,20,21]. Worthy of note is the approximately 10 million metric ton increase recorded between 2018 and 2021 (4 years), an increase that previously took about 13 years. With a shared mix of 452,000 tons of eggs in 2018, the South African poultry industry is not left behind. Despite the 10.2% shell content, these figures calls for a fast and decisive action especially when all other waste is put into context [22].

The concerning upward trajectory of waste production provides the basis for the concept of a circular economy. A circular economy aims to eliminate waste through the perpetual use of resources. This approach involves implementing practices such as reuse, sharing, repair, renovation, remanufacturing, and recycling to establish a closed system that minimizes the utilization of resources and the production of waste, pollution, and emissions [23,24]. Essentially, the idea is that all “waste” should become “food” for another process [25].

Various researchers have repeatedly exemplified the use of composites including eggshells, eggshell membranes, and metal nanoparticles. In construction, ES and ESM are particularly useful in full or partial replacement of aggregates in masonry applications, production of lightweight foamed concrete, aggregate stabilization, and power insulation [26,27,28,29,30,31,32,33].

In light of these use cases and many more, concrete structures keeps suffering from microbial attacks leading to their quick deterioration and consequently huge maintenance cost [5,34,35]. Some of the organisms involved in the biodeterioration of concrete structures include *Pseudomonas*, *Arthrobacter*, Algae, *Salmonella*, *E. coli*, and *Acidithiobacillus*. These organisms are usually found to attack concrete structures in waterlogged areas, sewers, splash zones, and reservoirs [36,37].

For this research, *Bacillus subtilis*, *Pseudomonas aeruginosa,* and industrially obtained waste activated sludge were selected. *Pseudomonas aeruginosa*, a gram-negative pathogen, is an opportunistic bacterium that is widely distributed and exhibits a high adaptability to different environmental conditions, including aquatic environments [38]. With a resistance rate of 33.9%, it displays significant resistance to antimicrobial agents, posing a considerable challenge in controlling its growth and spread, particularly in aquatic settings [39,40].

On the contrary, *Bacillus subtilis*, despite being gram-positive, demonstrates remarkable resilience against harsh conditions such as high temperatures, UV radiation, and γ-radiation. It can be found in various environments and readily adapts to thrive in diverse settings across the biosphere, ranging from soil to marine habitats [41,42]. Among different bacterial groups, *B. subtilis* exhibits the highest occurrence in hospital settings, and a substantial proportion of its strains are resistant to multiple antimicrobials. The ability of *B. subtilis* to withstand adverse conditions presents challenges for cleaning and disinfection efforts [43].

Waste-activated sludge (WAS) is a microbial biomass resulting from the dissolution of organic contaminants in municipal wastewater treatment facilities. Due to its mode of formation, WAS is a home to a consortium of aerobic and anaerobic microbial organisms. This consortium primarily encompasses eukaryotes, bacteria, archaea, and viruses, with bacteria being the predominant presence within the system. Some of the bacteria include *Proteobacteria, Bacteroidetes*, *Acidobacteria*, *Firmicutes*, and *Nitrospirae* [44,45,46].

As explained by Qi et al. (2020) [5], antimicrobial agents can either be inorganic or organic. Inorganic antimicrobial agents typically exhibit a prolonged lifespan and excellent resistance to high temperatures. However, they often come with side effects such as toxicity. On the other hand, organic antimicrobial agents demonstrate a clear bactericidal effect in a short period and offer a broad spectrum of activity against various pathogens. However, they tend to have poor resistance to high temperatures. An ideal option is, therefore, one that combines both properties.

Examples of inorganic antimicrobial agents that have been used in concrete are heavy metals, copper coating, zinc oxide, silver-loaded zeolite, Zeomighty, sodium tungstate, and silver nanoparticles. Organic antimicrobial agents include ConShield, ConBlock, eggshell, quats, and eggshell membranes [5,6,10,35,47].

It is on this premise that this paper presents a novel approach using a composite of eggshell membrane and silver nitrate or silver nanoparticles as microbial cell inhibition agents in cement mortar while optimizing the mechanical strength of the mortar.

## 2. Results and Discussion

### 2.1. Characterization of ESM and Mortar Composites

**Figure 1:**
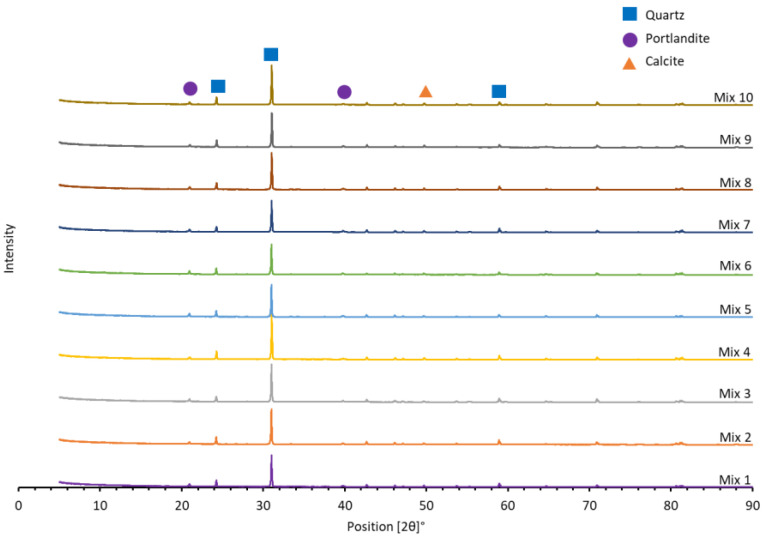
XRD mineralogy patter of the mortar mixes.

As observed in Figure 1, X-ray diffraction analysis implies that, despite the addition of Ag modified membranes to mixes 2 through 7, the mineralogy of the mortar mixes remains unchanged. This indicates that the produced cytotoxic mortar will have comparable characteristics to standard mortar. Quartz was the most predominant mineral followed by portlandite, calcite, mayenite, with traces of brownmillerite, larnite, and ettringite.

**Figure 2:**
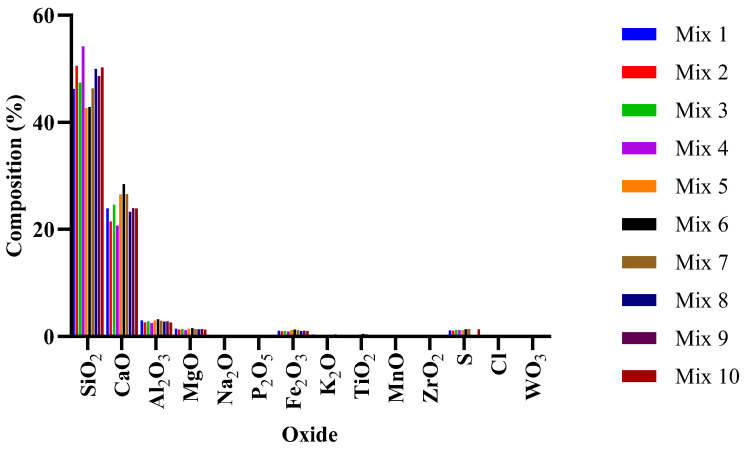
XRF oxide compositions of the different concrete mixes.

The oxide composition shown in Figure 2 follows a similar analogy as the XRD result with relatively constant composition across the mix. A two-way ANOVA comparison of the XRF data indicated that no significant difference between the compositions of the different mixes were confirmed (*p* = 0.9995) and that the variations between the results is most likely a result of random chance [48].

**Figure 3:**
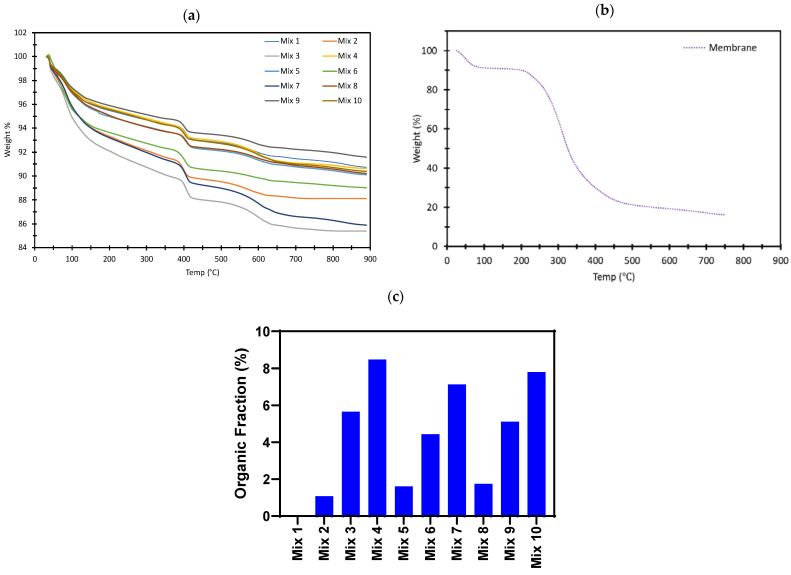
Thermogravimetric analysis: (**a**) Mixes 1 to 10; (**b**) Eggshell membrane; (**c**) Calculated organic fractions calculated using the method of Calvino et al., 2022 [49].

Furthermore, A TGA5500 thermogravimetric analyzer was used to observe the temperature stability of the mixes from 30 °C to 900 °C (Figure 3a). Thermogravimetric (TG), derivative thermogravimetry (DTG), and Differential Scanning Calorimetry (DSC) analyses were employed (Supplementary Figure S1). All mix samples experienced two stages of weight loss. The initial loss, which was gentle, occurred over a wide temperature range of 30 °C and 150 °C. This loss can be attributed to loss of moisture content. The second significant loss was very abrupt and quickly occurred between 390 °C and 420 °C due to thermal decomposition. All mixes were relatively thermally stable compared to the control mix, mix 1 with a maximum difference of 5% weight loss due to the presence of shell membrane. The eggshell membrane was also tested as illustrated in Figure 3b and turns completely into ash after 300 °C, leaving approximately 10% weight at >700 °C. Using the methods proposed by Calvino et al. (2022) [49], the organic fractions of the different mortar mixes were calculated and are shown in Figure 3c. The results clearly correspond to the expected compositions of the mortar mixes as presented in Table 1.

**Figure 4:**
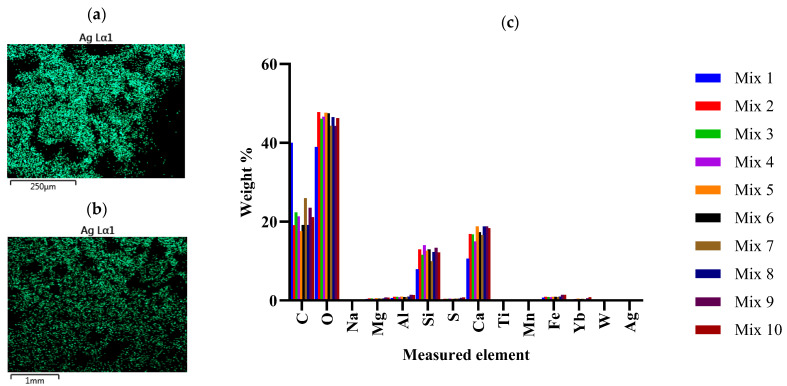
SEM EDS Ag distribution map: (**a**) AgNO_3_/ESM Mortar (MIX 4); (**b**) AgNPs/ESM Mortar (MIX 7); (**c**) The elemental compositions of the different mortar mixes (as measured using EDS).

SEM-EDS analysis was previously conducted and confirmed the adsorption of AgNPs and AgNO_3_ on the membrane, notably both AgNO_3_ and AgNPs were uniformly absorbed onto the membrane’s surface [50]. The porous fibril structure, known for its ability to enhance ESM’s absorption capacity [51], was found to be effective. In the current study the retention of the Ag within the mortar mix was confirmed (Figure 4a,b and Table S1). Ag EDS measurements of the mixes supported the presence of Ag in mixes 3, 4, 7, and 8, with increasing Ag content measured. No Ag was measured by EDS in mixes 2 and 5, likely due to the detection limit of the machine—the Ag content of mixes 3 and 6 were only 0.06% and 0.05%, respectively.

The material exhibited relatively similar macroscopic features with limited differences observable from the SEM micrograph (Figure S2) and the elemental compositions for the different mixes as measured using EDS (Figure 4c and Figure S2) were found to exhibit no significant differences when applying a two-way ANOVA analysis; the *p* value was found to be greater than 0.9999.

### 2.2. Antimicrobial Activity of Mortar Composite

**Figure 5:**
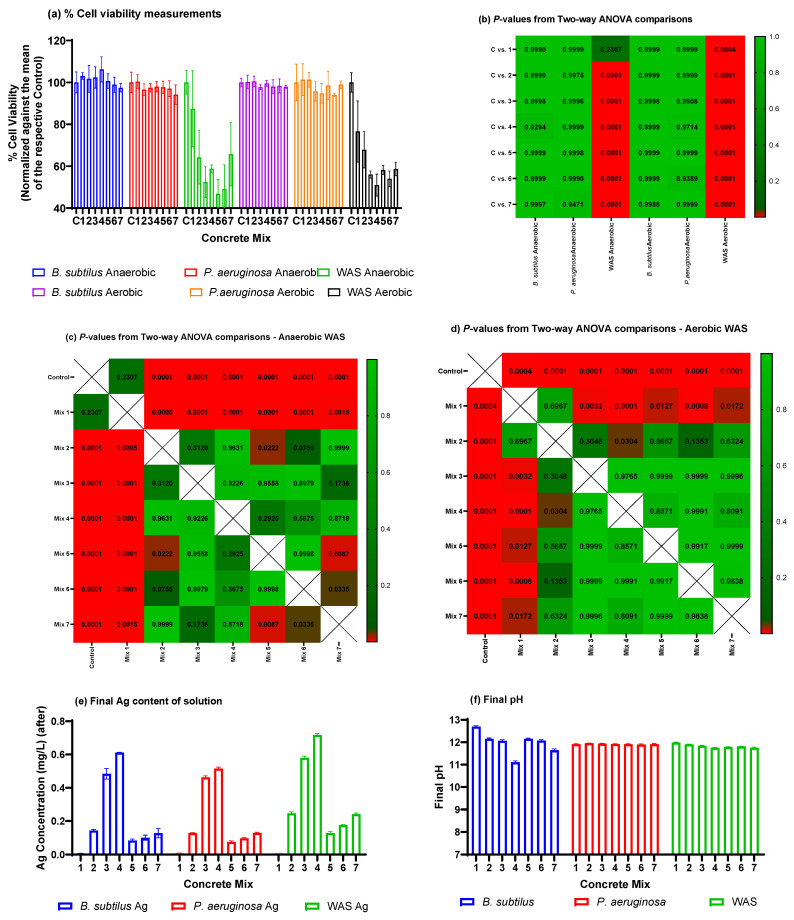
(**a**) % cell viability measurements for the concrete mixture experiments normalized against the means of the control experiment for each microbial culture; (**b**–**d**) *p*-values from Two-way ANOVA comparisons between the % cell viabilities for the control and different concrete mixes, the anaerobic WAS and aerobic WAS runs, respectively; (**e**) The Ag concentrations of the aqueous solutions for each concrete mix exposed to different microbial cultures; (**f**) The final pH of the respective concrete mixes exposed to different microbial cultures.

The effects of Ag-impregnated mortar composites are illustrated in Figure 5a. Figure 5b summarizes the *p*-values obtained from the two-way ANOVA comparisons (Tukey’s multi-comparison test) between the control group and mixes 1−7. Notably, the most significant differences were observed when introducing Ag/ESM mortar mixes to the waste activated sludge (WAS). The *p*-values indicated highly significant differences (*p* < 0.0001) between the control and mixes 3−7 for anaerobic runs, and between the control and mixes 2−7 for aerobic runs. However, the comparison between the control and mix 2 in the anaerobic run did not show significant differences (*p* = 0.2307). The most pronounced cytotoxic effect of the antimicrobial composites on WAS occurred under anaerobic conditions, resulting in a cell viability of 36.8 ± 6.1% for AgNPs composite mix 5. Under aerobic conditions, the cytotoxic effect was observed with mix 4, resulting in a cell viability of 51.0 ± 4.8% for WAS.

Furthermore, in the anaerobic WAS run, significant differences were observed between mixes 1 and mixes 2 to 7, mixes 2 and 5, mixes 5 and 7, and mixes 6 and 7 (Figure 5c). In the case of aerobic runs, significant differences were measured between mixes 1 and 3 to 7, and mixes 2 and 4 (Figure 5d). However, for the runs involving *B. subtilis* and *P. aeruginosa*, no significant differences were observed between the control group and any of the mortar mixes in either the anaerobic or aerobic runs.

To explain these inhibitory results, Figure 5e displays the silver concentrations measured using Atomic Absorption Spectroscopy (AAS) after exposure to *B. subtilis*, *P. aeruginosa*, and WAS. The measured Ag results were consistent with the initially dosed Ag/ESM composite content of the mortar mixes (i.e., 1%, 2%, and 5% replacements, respectively). Notably, the Ag concentrations in solution were relatively low, ranging from approximately 0.1 mg/L to 0.7 mg/L for AgNO_3_-impregnated mortar mixes and from around 0.07 mg/L to 0.2 mg/L for AgNPs-impregnated mortars.

It is important to mention that the minimum inhibitory concentration for AgNO_3_ was reported to be at least 1 mg/L for B. subtilis [52] and between 8 and 16 mg/L for *P. aeruginosa* [53], while for AgNPs it ranged between 2 and 5 mg/L for both B. subtilis and *P. aeruginosa* [52,54]. In the case of WAS, the efficacy of Ag^+^ and AgNPs was significantly more pronounced, with Ag^+^ concentrations as low as 0.5 mg/L (from AgNO_3_) and AgNPs at 0.2 mg/L reported to inhibit enriched nitrifying bacteria by up to 50% and 60%, respectively [55,56].

The pH levels of the individual bacterium cultures were initially recorded as 6.82, 7.01, and 6.61 for *B. subtilis*, *P. aeruginosa*, and WAS, respectively, before exposure. After exposure, all culture pH values rose to around 12 (see Figure 5f). This shift in pH has been shown to impact WAS systems, leading to increased concentrations of volatile fatty acids (VFA), ammonia, and phosphate, while suppressing methane production, indicating the disruption of normal biological function under these high pH conditions [57]. In contrast, *B. subtilis* and *P. aeruginosa* demonstrated greater resistance to pH changes [58,59]. While the possibility that the decreased Ag content in the solutions could be attributed to Ag precipitation at the elevated pHs, Aina et al. (2023) [50] demonstrated that the Ag in the Ag/ESM composites existed primarily in either the elemental or oxidized forms. This would result in the Ag content in the liquid matrix being less susceptible to removal at elevated pH values [60].

The limited impact of the different mortar mixes on *B. subtilis* and *P. aeruginosa* cultures can be attributed to the relatively low Ag concentrations in solution (below inhibitory concentrations) and the cultures’ resistance to elevated pH levels. In contrast, the significant inhibition of WAS by the mortar mixes, including the concrete-only mortar (mix 1) in aerobic runs, may be attributed to the presence of Ag^+^ or AgNPs in the mixture, which has been shown to affect WAS, along with the elevated pH levels recorded in the solutions. These findings suggest potential applications of antimicrobial mortar mixtures, even at low Ag/ESM concentrations, within sewage carriage systems. However, it is important to note that these applications may not effectively control *B. subtilis* or *P. aeruginosa* contamination.

### 2.3. Mechanical Strength Characteristics of Mortar Composite

**Figure 6:**
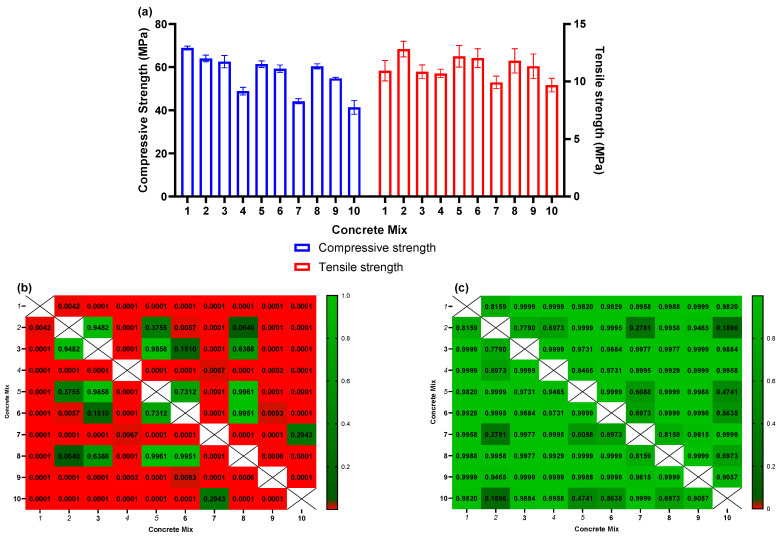
(**a**) Comparisons of compressive and tensile strengths for the different concrete mixes; (**b**,**c**) The *p*-values obtained from the two-way ANOVA analyses comparing each concrete mix to each other concrete mix as related to the compressive and tensile strengths, respectively.

After a curing period of 28 days, the hardened cubes underwent testing to assess both compressive and tensile strength, as indicated in Figure 6a. In terms of compressive strength, the control mix displayed an average value of 68.9 ± 0.9 MPa (mean ± standard deviation). However, the composite mixes exhibited a noteworthy decline in compressive strength, ranging from 64.0 ± 1.6 MPa for mix 2 to 41.4 ± 3.2 MPa for mix 10. Statistical analyses using two-way ANOVA further confirmed the significance of these differences in comparison to the baseline (mix 1). This decline aligns with prior research findings that have reported reduced compressive strength as the proportion of eggshell powder increases [26,27,61]. Additionally, there were no statistically significant differences observed among mixes with similar eggshell fractions (2 vs. 5 vs. 8, 3 vs. 6 vs. 8, and 7 vs. 10), underscoring that the primary factor contributing to the compressive strength decrease is the content of eggshell powder (ESM).

In contrast, the tensile strength of the mixes did not exhibit significant differences when compared to the control mix (Figure 6c). The control mix registered an average tensile strength of 10.9 ± 0.9 MPa, while the remaining mixes reported tensile strengths ranging from 12.8 ± 0.7 to 9.7 ± 0.6 MPa. Analyzing the tensile strengths across the various mixes through two-way ANOVA revealed no statistically significant distinctions (at α = 0.05) between any of the mixtures.

The most favorable combinations in this study were blends 3, 5, and 6. Blend 3 contained 2% ESM/AgNO3, blend 5 contained 1% ESM/AgNPs composite, and blend 6 contained 2% ESM/AgNPs composite. None of these blends caused a noteworthy alteration in the cell viability of either *B. subtilis* or *P. aeruginosa* when compared to the control. However, when assessing the cell viability of waste activated sludge (WAS) in anaerobic systems, it was found that blends 3, 5, and 6 resulted in viabilities of 52.4% ± 7.4%, 46.8% ± 6.9%, and 49.1% ± 11.6%, respectively, in contrast to the control. Under aerobic conditions, the cell viability for these three blends was 56.0% ± 1.7%, 58.2% ± 2.1%, and 54.0% ± 3.7%, respectively. Statistical analysis using two-way ANOVA revealed no significant differences at the α = 0.05 level among these results.

Importantly, the proposed optimal blends exhibited compressive strengths of 62.6 MPa ± 2.8 MPa, 61.4 MPa ± 1.5 MPa, and 69.3 MPa ± 1.54 MPa, respectively, compared to 68.9 ± 0.9 MPa for the control without eggshell, representing an approximate 10% reduction in strength. Additionally, the tensile strengths of these blends were 10.9 MPa ± 0.6 MPa, 12.2 MPa ± 0.9 MPa, and 12.0 MPa ± 0.8 MPa, respectively, which did not exhibit statistically significant changes at the α = 0.05 level when compared to the control’s tensile strength of 10.9 ± 0.9 MPa.

## 3. Materials and Methods

### 3.1. Preparation of Ag-Modified Eggshell membranes (Ag-EMs)

The Ag-EMs were created using the method previously outlined [50]. The procedure was as follows:

Eggshells were procured from nearby restaurants around the University of Pretoria. These shells were promptly washed after collection and then dried at 60 °C for one hour. Following decontamination, the shells were stored in plastic bags until the separation phase. To aid membrane separation, the shells were soaked in 1 mol/L acetic acid for 17 min. The extraction of the ESM was then carried out manually [51]. All extracted membranes underwent a rinsing process with deionized water, subsequent drying, and storage.

To modify the ESM, silver nitrate (AgNO_3_), and silver nanoparticles (AgNPs) were employed. AgNPs were synthesized through the chemical reduction of AgNO_3_ (Sigma-Aldrich, Johannesburg, South Africa, ≥99%, 169.87 g/mol). The chemical reduction process, as detailed in [17,62], employed NaBH_4_ (Sigma-Aldrich Johannesburg, South Africa, ≥98%, 37.83 g/mol) as the reducing agent and trisodium citrate dihydrate (Na_3_C_6_H_5_O_7_·2H_2_O) (Fisher Scientific, Johannesburg, South Africa, ≥99%, 294.10 g/mol) as a ligand. In the initial steps, a solution of Na_3_C_6_H_5_O_7_·2H_2_O (0.01 M, 100 mL), AgNO_3_ (0.01 M, 50 mL), and NaBH_4_ (0.01 M, 50 mL) was prepared using deionized water. Subsequently, 1 mL of AgNO_3_, 1 mL of Na_3_C_6_H_5_O_7_·2H_2_O, and 20 mL of deionized water were combined in a 100 mL beaker, which was then positioned in an ice bath. Stirring the solution with a magnetic stirrer for 5 min yielded a nano silver solution. NaBH_4_ was then added dropwise until the suspension turned a bright yellow, and the mixture was continuously stirred for two hours to guarantee a complete reduction reaction.

ESMs of varying particle sizes, ranging from 1 mm to 5 mm (obtained by sieving), were subjected to adsorption with both synthesized AgNPs and AgNO_3_. This adsorption process involved introducing the dried ESM into 40 mL glass Polytops containing diluted solutions of AgNPs or AgNO_3_. These setups were subsequently placed on an oscillator at 25 °C and a pH of 6 for 48 h to facilitate the adsorption process. The adsorbed loadings of AgNPs and AgNO_3_ aligned with the maximum adsorption capacities reported in Aina et al. (2023) [50], approximately 0.6 mg/g and around 16 mg/g, respectively.

### 3.2. Preparation of Cement Mortar

The mortar was formulated in compliance with the specifications of the European Committee for Standardization (CEN), as outlined in EN 196-1:2005 [63], and adhered to the guidelines provided by the South African Bureau of Standards, as stated in SANS 50196-1:2006 [64].

**Figure 7:**
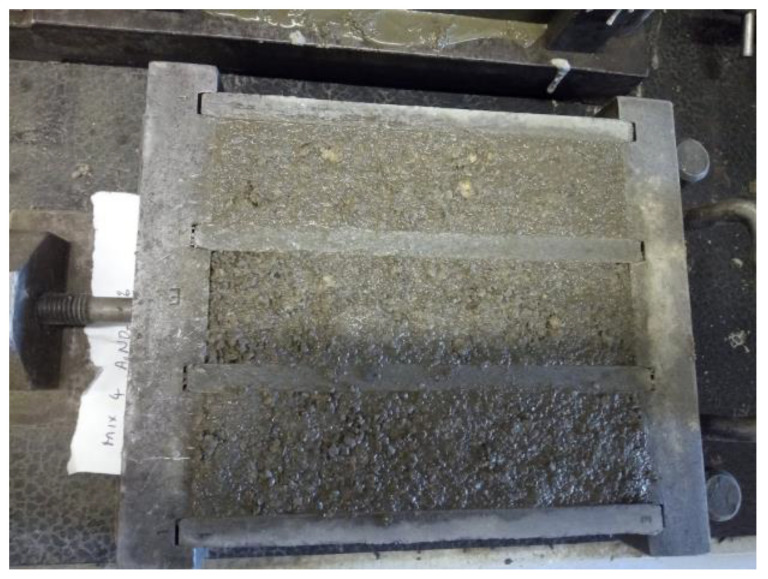
Mixed Mortar in Prism Mold.

For the control sample (Mix 1), the preparation involved weighing and mechanically mixing one part of 52.5R cement, three parts of CEN standard sand, and half a part of water for a duration of 150 s. To create variation, nine additional mixes were produced by substituting a portion of the cement content with 1%, 2%, and 5% of ESM and modified ESM, as detailed in Table 1. All the mixtures underwent mechanical mixing for a period of 150 s. Subsequently, the mixed mortar was placed into a series of three prism molds measuring 40 mm × 40 mm × 160 mm (Figure 7) and left for 24 h before being demolded. Following demolding, the specimens were immersed in water for curing over a period of 28 days.

### 3.3. Characterization of ESM Modified Mortar

X-ray diffraction (XRD) was used to understand the mineralogy of the mortar mixes while their oxide composition was determined with the use of X-ray fluorescence (XRF) analysis. During the XRD analysis, the samples were prepared utilizing the standardized PANalytical backloading system, which ensures a nearly random distribution of particles. Analysis of the samples was performed using a PANalytical X’Pert Pro powder diffractometer configured in θ–θ mode, equipped with an X’Celerator detector. The instrument employed Fe-filtered Co-Kα radiation (λ = 1.789 Å) along with variable divergence and fixed receiving slits. To determine the mineralogy, the measured diffraction pattern was compared to patterns in the ICSD database, and the best-fitting pattern was selected using X’Pert Highscore plus 5.1 software (Malvern Panalytical Ltd., Malvern, UK). The relative amounts of each phase, expressed as weight percentages of the crystalline portion, were estimated using the Rietveld method with X’Pert Highscore plus software.

To carryout XRF analysis, 10−30 g of powdered sample were combined with 20 drops of Moviol (PVA) and pressed with a force of 10 tons. The Thermo Fisher ARL Perform’X Sequential XRF instrument, along with the Uniquant 5 software (Thermo Fisher Scientific, Waltham, MA, USA), was utilized for the analyses. The software examined all elements in the periodic table from sodium (Na) to uranium (U), but only reported the elements detected above the specified limits. The reported values were not normalized since no LOI (Loss on Ignition) process was conducted to determine changes in crystal water and oxidation states. A standard sample material was prepared and analyzed using the same procedure as the mix samples.

The morphology of all mortar mix was studied in a Zeiss Ultra PLUS FEG scanning electron microscope (SEM). Samples were dried before being sputter-coated with carbon in a Quorum Q150T ES (Quorum Technologies, Lewes, UK) coater for imaging. SEM and energy dispersive X-ray analysis (EDX) was also conducted to understand the distribution and elemental composition of each mix.

Thermal analysis (TGA) was conducted to examine the change in mass with an increase in temperature of each mortar mix. This was investigated using a TGA5500 thermogravimetric analyzer to observe the impact of elevated temperatures on each mortar mix. The alteration in mass was assessed as the temperature rose from ambient temperature to 900 °C, with a heating rate of 10 °C/min in the presence of nitrogen. Differential scanning calorimetry (DSC) and Differential Thermal Analysis (DTA) measurements were also carried out using the TA Instruments in the presence of nitrogen at a temperature rate of 10 °C/min.

### 3.4. Antimicrobial Activity of ESM Modified Mortar

The antimicrobial activities against *Bacillus subtilis* (BS) and *Pseudomonas aeruginosa* (PA) and waste activate sludge (WAS) was determined using MTT assay. MTT assay, which stands for 3-(4,5-dimethylthiazolyl-2)-2,5-diphenyltetrazolium bromide assay, is a reliable and sensitive colorimetric method employed to assess the metabolic activity of cells. This assay involves the reduction of a tetrazolium dye using specific bacterial enzymes, resulting in the formation of an insoluble purple compound called formazan. The concentration of formazan is determined by measuring its absorbance using a spectrometer in the wavelength range of 500 to 700 nm. As the number of viable bacteria increases, the concentration of formazan also increases, leading to a more pronounced purple coloration and higher absorbance values [65,66,67].

To grow overnight cultures of both bacteria, nutrient broth was used at a temperature of 37 °C while shaking at 150 rpm until an optical density of 0.4 was reached. The cultures were then subjected to centrifugation, and the supernatant was washed twice using distilled water before being resuspended in distilled water. The optical density was then adjusted to approximately 1.

Ground mortar was exposed to both bacteria in aerobic and anaerobic conditions. For the aerobic test, 1 mL bacteria was exposed to 2 g of mortar in 24 mL broth in an incubator at 37 °C and 200 rpm for 4 h using 100 mL dark vials. A similar procedure was used for the anaerobic test with the addition of sodium nitrate.

At the end of the fourth hour, 2.5 mL MTT was added to the medium and further exposed for 3 h at 160 RPM in the absence of light. The absorbance was measured at 700 nm using a VWR UV-1600PC spectrophotometer. A stock solution of 5 mg/mL of MTT in 0.1 M PBS (pH 7) was used. Mixes 8,9, and 10 were not tested due to the lack bacteria inhibition by eggshell membrane [50].

pH was with the GOnDO PL-700ALS bench top meter and its pH probe. An atomic absorption spectrometer (Perkin Elmer AAnalyst 400, Waltham, MA, USA) was used to measure silver concentration in the solution after exposure with an SJ hollow silver lamp.

### 3.5. Mechanical Strength Test

The density of each prism was calculated by weighing each in air and water and using the formular:

(1) $$\frac{\rho_{m}}{\rho_{w}}=\frac{W\left(\mathrm{air}\right)}{W\left(\mathrm{air}\right)-W\left(\mathrm{water}\right)}$$

$\rho_{m}$ density of mortar in g/cm^3^.
$\rho_{w}$ density of water in g/cm^3^ = 1 g/cm^3^.
W(air) is weight in air in g.
W(water) is weight in water in g.

Flexural strength test was carried out in triplicate using a Versa Tester using the 3-point loading system. The load was vertically applied with the loading roller at a rate of 50 N/s until fracture. The flexural strength was calculated in MPA using

(2) $$R_{f}=\frac{1.5\times F_{f}\times l}{b^{3}}$$

*R_f_* is the flexural strength, in megapascals.
*b* is the side of the square section of the prism, in millimeters.
*F_f_* is the load applied to the middle of the prism at fracture, in newtons.
*l* is the distance between the supports, in millimeters.

Both half of each prism used for flexural test was kept and used for compressive testing. An AutoMax UTest material testing machine was used to conduct the compressive test at a loading rate of 2400 N/s until fracture. The compressive strength was calculated in MPA using

(3) $$R_{c}=\frac{F_{c}}{1600}$$

where *R_c_* is the compressive strength, in megapascals, and *F_c_* is the maximum load at fracture, in newtons.

## 4. Conclusions

The findings presented have further underscored the advantages of composite materials and composite nanomaterials. A composite cytotoxic mortar was successfully manufactured, possessing a mineral composition akin to standard mortar. However, it exhibited an exceptional antimicrobial property, resulting in up to a 50% reduction in cell viability in wastewater, all while marginally enhancing tensile strength and showing only a slight reduction in compressive strength.

From these results it can be concluded that mixes 3, 5, and 6 provided the optimal balance between antimicrobial properties (there were no significant differences for mixes 3 to 7 measured in terms of antimicrobial activities), and the loss in compressive/tensile strength of the concrete.

In the context of promoting a circular economy, the utilization of eggshell and its membrane has further demonstrated their practicality. The implementation of this antimicrobial composite holds the potential to not only mitigate waste management challenges but also to safeguard concrete structures against microbial deterioration.

## Figures and Tables

**Table 1:** Mix Design.

Mix No	Membrane	% Replacement	Membrane (g)	Cement (g)	Sand (g)	Water (g)
1	-	0	-	500	1500	250
2	AgNO_3_/ESM	1	5	495	1500	250
3	AgNO_3_/ESM	2	10	490	1500	250
4	AgNO_3_/ESM	5	25	475	1500	250
5	AgNPs/ESM	1	5	495	1500	250
6	AgNPs/ESM	2	10	490	1500	250
7	AgNPs/ESM	5	25	475	1500	250
8	ESM	1	5	495	1500	250
9	ESM	2	10	490	1500	250
10	ESM	5	25	475	1500	250

## Data Availability

The data presented in this study are available on request from the corresponding author.

## Supplementary Materials

The following supporting information can be downloaded at: https://www.mdpi.com/article/10.3390/ijms242015463/s1.
