# Supplementary material for: Cytotoxic-Ag-Modified Eggshell Membrane Nanocomposites as Bactericides in Concrete Mortar
Source: Int J Mol Sci. 2023 Oct 23;24(20):15463. doi: 10.3390/ijms242015463 (PMC10607369; doi:10.3390/ijms242015463)
Supplement: Supplementary file 1 [file ijms-24-15463-s001.zip › ijms-2640484-supplementary.pdf]

International Journal of *Molecular Sciences*

MDPI

*Article*

# Cytotoxic-Ag-Modified Eggshell Membrane Nanocomposites as Bactericides in Concrete Mortar

**Samuel Tomi Aina [1], Hilda Dinah Kyomuhimbo [1], Barend Du Plessis [1], Vuyo Mjimba [2], Nils Haneklaus [3] and Hendrik Gideon Brink [1,*]**

1 Department of Chemical Engineering, University of Pretoria, South Africa; samuel.aina@tuks.co.za (S.T.A.); u21830658@tuks.co.za (H.D.K.); barend.duplessis@up.ac.za (B.D.P.)
2 Human Sciences Research Council, Pretoria, South Africa; vmjimba@hsrc.ac.za
3 Td Lab Sustainable Mineral Resources, University for Continuing Education Krems, Dr.-Karl-Dorrek-Straße 30, 3500 Krems, Austria; nils.haneklaus@donau-uni.ac.at

* Correspondence: deon.brink@up.ac.za

## Supplementary Material

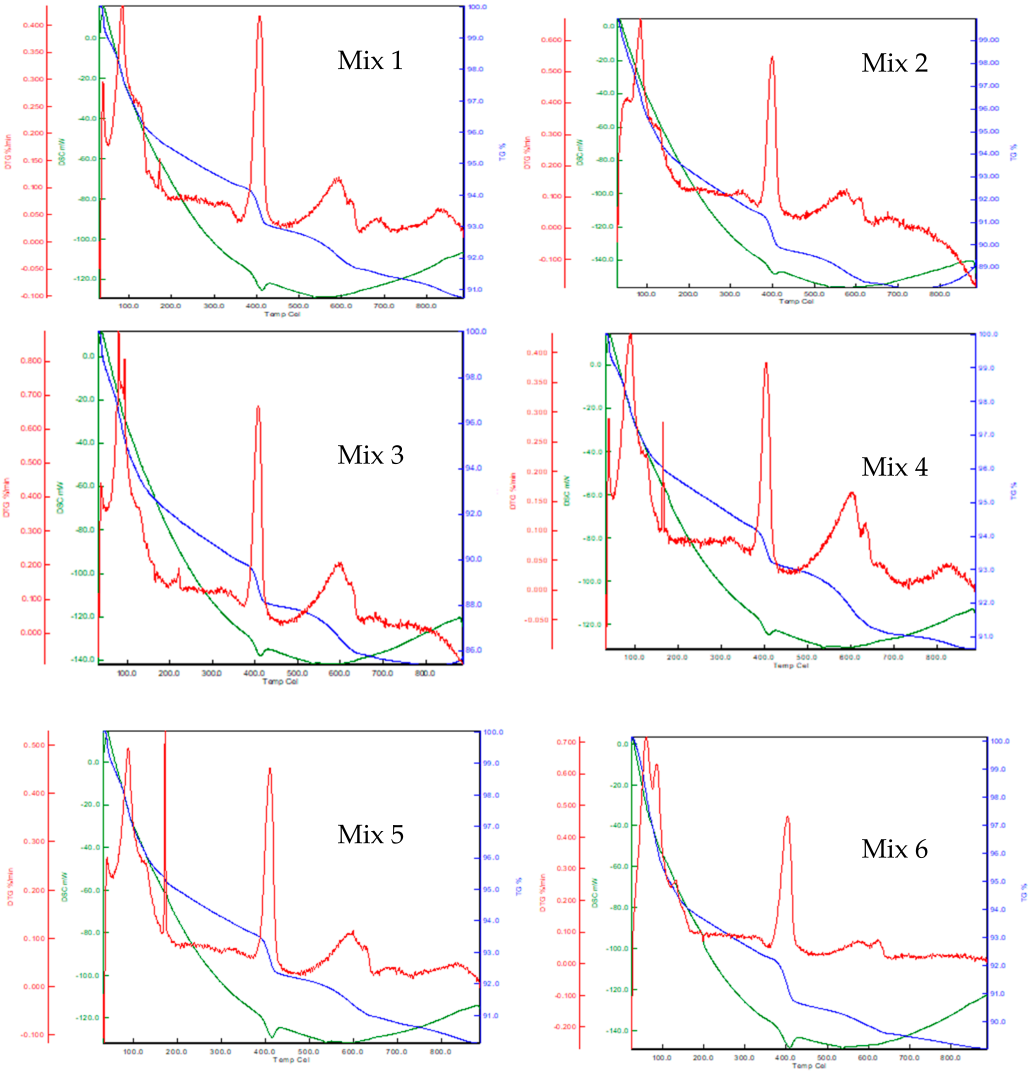

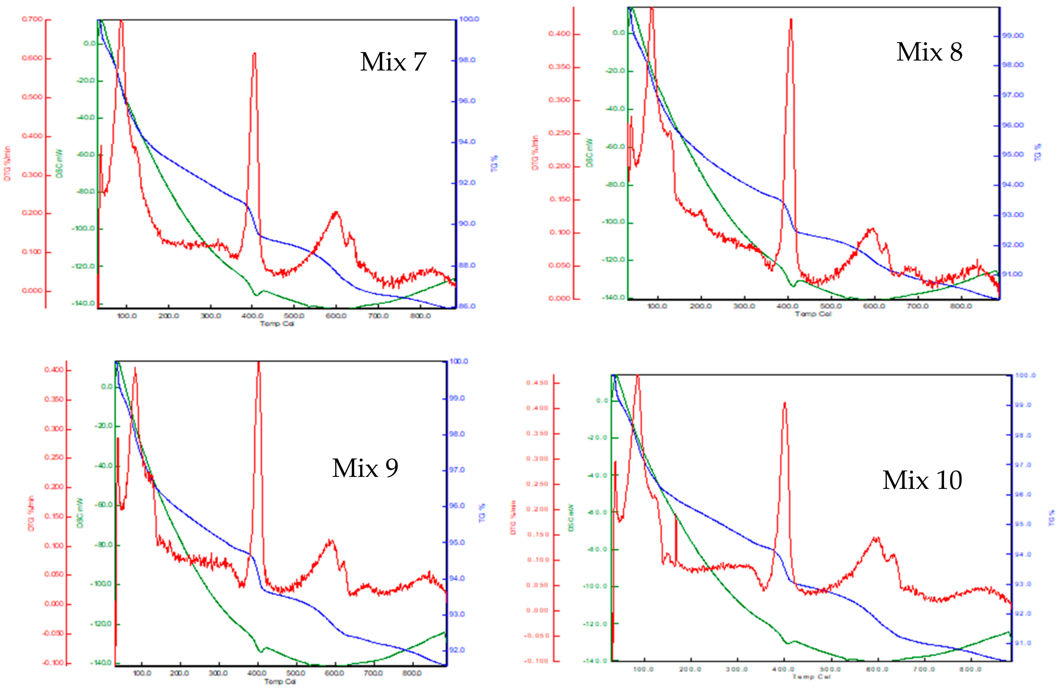


**Figure S1.** TG, DTG, and DSC analysis of concrete mixes. A to J for mix 1 to 10 respectively.

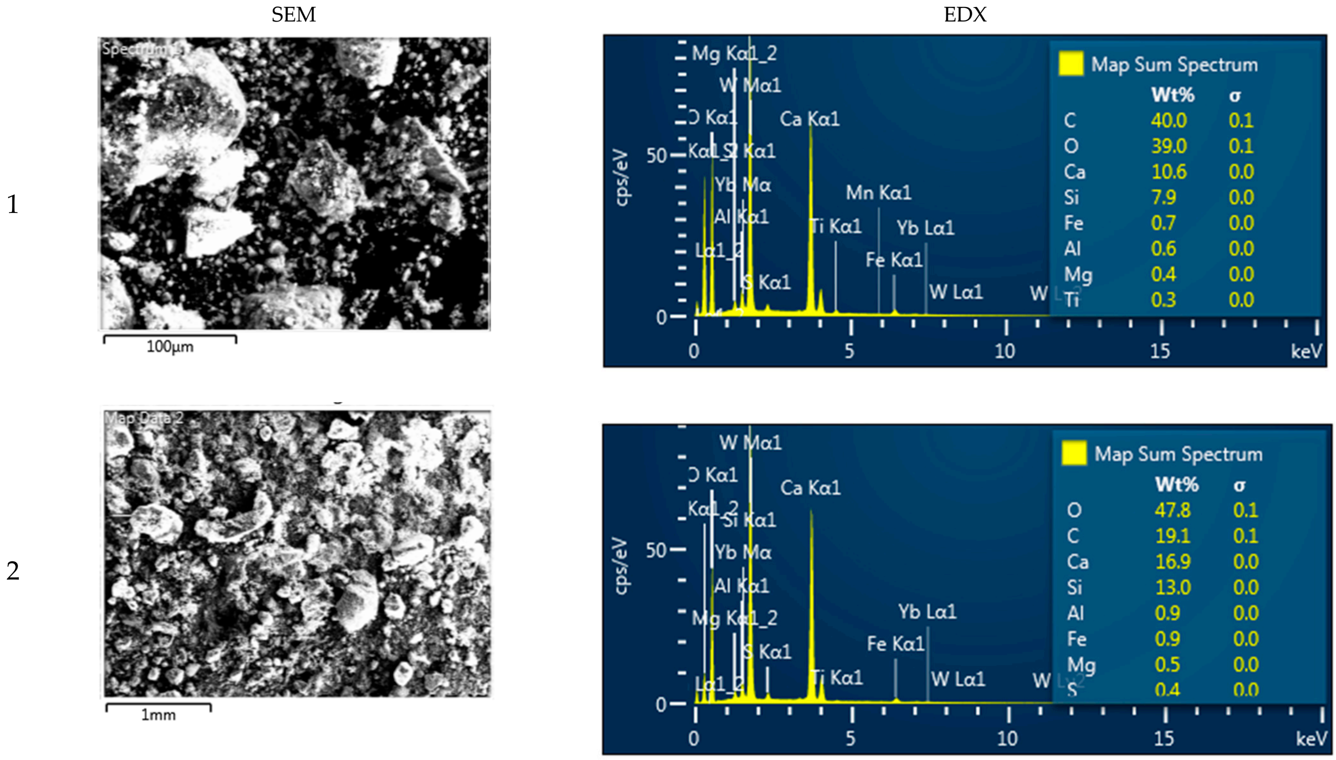

3

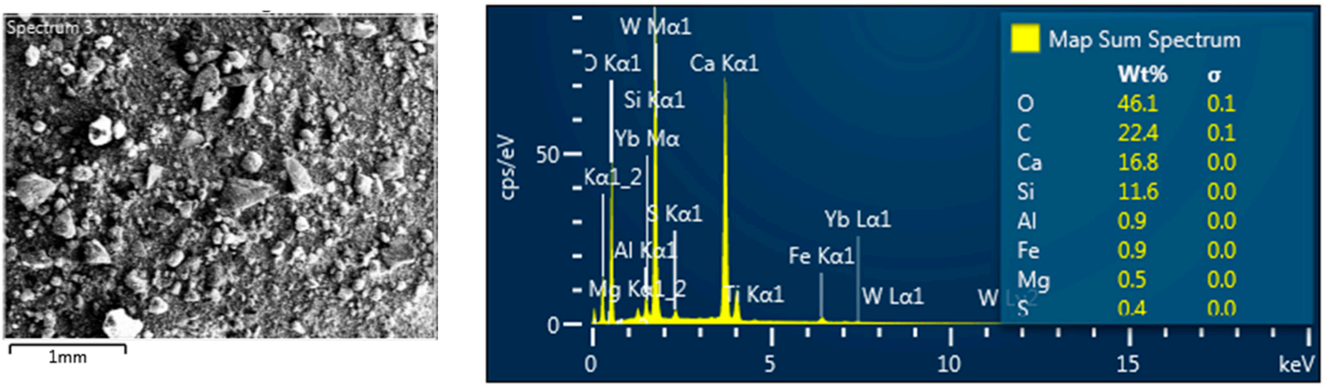
Spectrum 3
1mm
W Mα1
O Kα1
Ca Kα1
Si Kα1
Yb Mα
Kα1_2
S Kα1
Al Kα1
Mg Kα1_2
Ti Kα1
Fe Kα1
Yb Lα1
W Lα1
cps/eV
50
0
0
5
10
15
keV
Map Sum Spectrum
Wt% σ
O 46.1 0.1
C 22.4 0.1
Ca 16.8 0.0
Si 11.6 0.0
Al 0.9 0.0
Fe 0.9 0.0
Mg 0.5 0.0
S 0.4 0.0

4

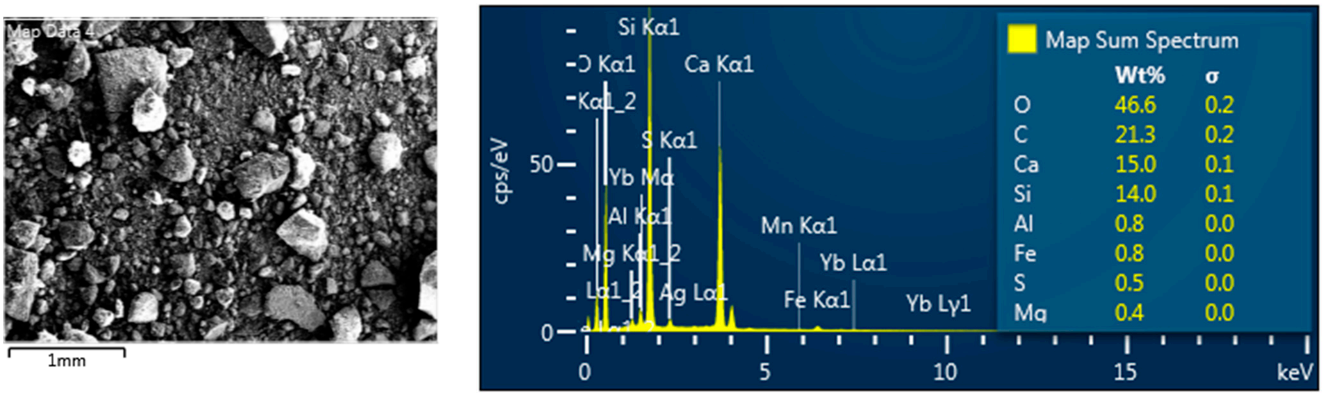
Map Data 4
1mm
Si Kα1
O Kα1
Ca Kα1
Kα1_2
S Kα1
Yb Mα
Al Kα1
Mg Kα1_2
Lα1_2
Ag Lα1
Mn Kα1
Yb Lα1
Fe Kα1
Yb Lγ1
cps/eV
50
0
0
5
10
15
keV
Map Sum Spectrum
Wt% σ
O 46.6 0.2
C 21.3 0.2
Ca 15.0 0.1
Si 14.0 0.1
Al 0.8 0.0
Fe 0.8 0.0
S 0.5 0.0
Mg 0.4 0.0

5

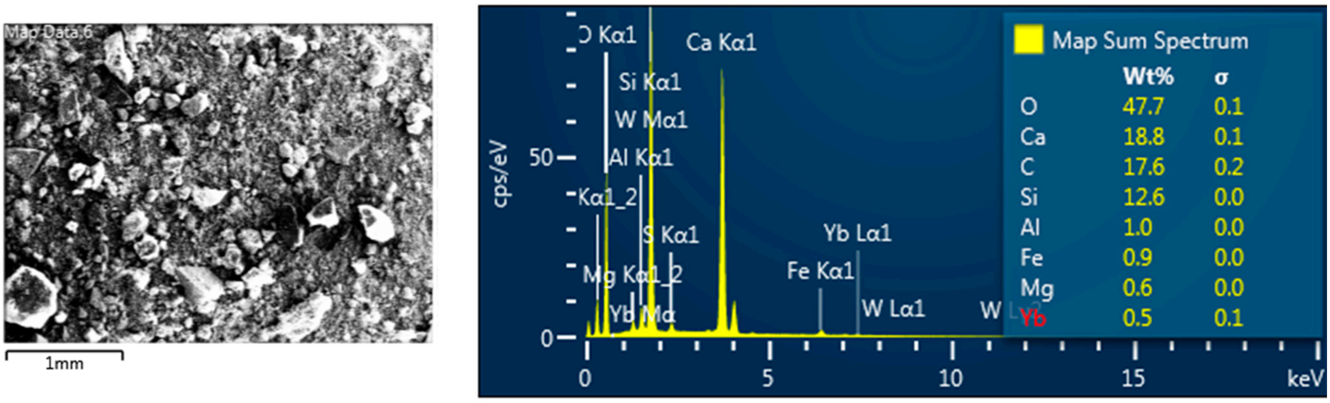
Map Data 6
1mm
O Kα1
Ca Kα1
Si Kα1
W Mα1
Al Kα1
Kα1_2
S Kα1
Mg Kα1_2
Yb Mα
Yb Lα1
Fe Kα1
W Lα1
cps/eV
50
0
0
5
10
15
keV
Map Sum Spectrum
Wt% σ
O 47.7 0.1
Ca 18.8 0.1
C 17.6 0.2
Si 12.6 0.0
Al 1.0 0.0
Fe 0.9 0.0
Mg 0.6 0.0
Yb 0.5 0.1

6

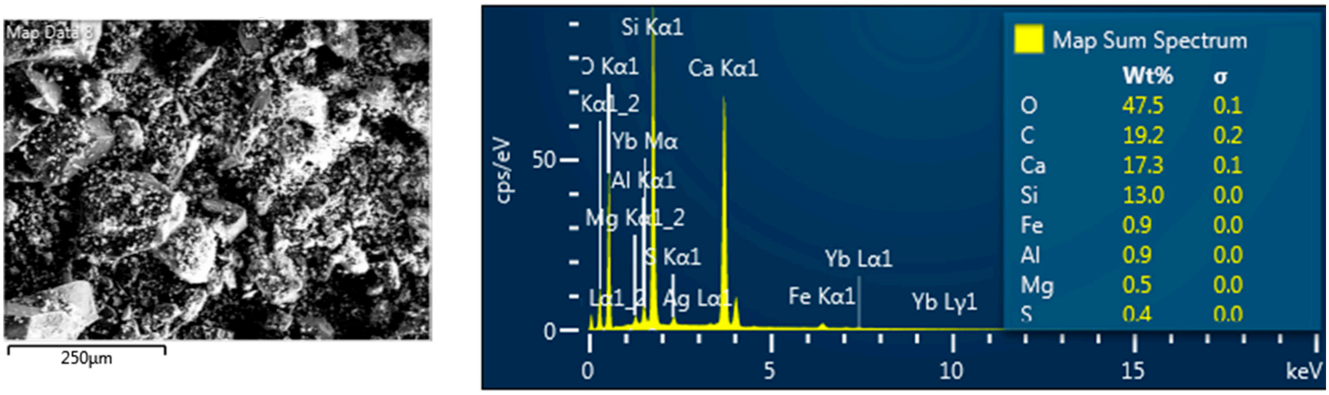
Map Data 8
250μm
Si Kα1
O Kα1
Ca Kα1
Kα1_2
Yb Mα
Al Kα1
Mg Kα1_2
S Kα1
Lα1_2
Ag Lα1
Yb Lα1
Fe Kα1
Yb Lγ1
cps/eV
50
0
0
5
10
15
keV
Map Sum Spectrum
Wt% σ
O 47.5 0.1
C 19.2 0.2
Ca 17.3 0.1
Si 13.0 0.0
Fe 0.9 0.0
Al 0.9 0.0
Mg 0.5 0.0
S 0.4 0.0

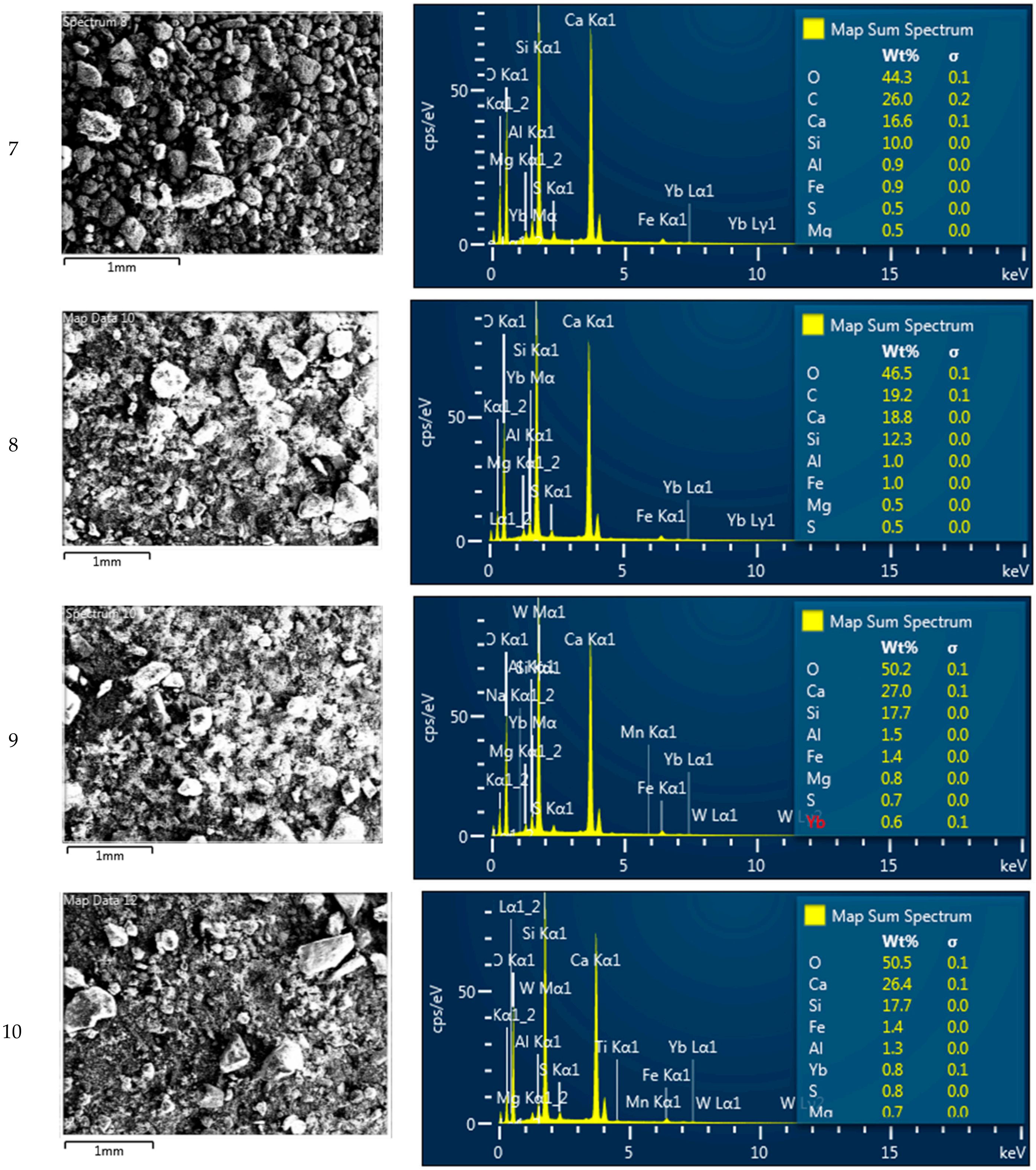


**Figure S2.** SEM Images and corresponding EDX profiles of mortar mixes.

**Table S1.** EDS percentage elemental composition of mortar mix

| Mix/Wt% | 1 | 2 | 3 | 4 | 5 | 6 | 7 | 8 | 9 | 10 |
|---|---|---|---|---|---|---|---|---|---|---|
| C | 40.02 | 19.1 | 22.36 | 21.3 | 17.59 | 19.19 | 25.96 | 19.19 | 23.51 | 21.14 |
| O | 38.96 | 47.81 | 46.14 | 46.64 | 47.67 | 47.47 | 44.31 | 46.53 | 44.25 | 46.28 |
| Na | 0 | 0 | 0 | 0 | 0 | 0 | 0 | 0 | 0.06 | 0 |

| | | | | | | | | | | |
|---|---|---|---|---|---|---|---|---|---|---|
| Mg | 0.36 | 0.51 | 0.51 | 0.43 | 0.56 | 0.47 | 0.46 | 0.52 | 0.77 | 0.71 |
| Al | 0.62 | 0.94 | 0.91 | 0.82 | 0.99 | 0.85 | 0.88 | 0.99 | 1.45 | 1.35 |
| Si | 7.91 | 12.99 | 11.55 | 14 | 12.59 | 12.98 | 9.96 | 12.25 | 13.36 | 12.16 |
| S | 0.25 | 0.4 | 0.42 | 0.45 | 0.43 | 0.42 | 0.48 | 0.46 | 0.69 | 0.76 |
| Ca | 10.62 | 16.86 | 16.75 | 14.96 | 18.76 | 17.33 | 16.65 | 18.76 | 18.76 | 18.33 |
| Ti | 0.29 | 0.13 | 0.15 | 0 | 0 | 0 | 0 | 0 | 0 | 0.26 |
| Mn | 0.04 | 0 | 0 | 0.05 | 0 | 0 | 0 | 0 | 0.07 | 0.09 |
| Fe | 0.73 | 0.92 | 0.87 | 0.8 | 0.94 | 0.92 | 0.86 | 0.97 | 1.44 | 1.44 |
| Yb | 0.2 | 0.34 | 0.35 | 0.36 | 0.47 | 0.37 | 0.42 | 0.33 | 0.6 | 0.82 |
| W | 0 | 0 | 0 | 0 | 0 | 0 | 0 | 0 | 0 | 0 |
| Ag | 0 | 0 | **0.06** | **0.2** | 0 | **0.05** | **0.3** | 0 | 0 | 0 |
| Total: | 100 | 100 | 100 | 100 | 100 | 100 | 100 | 100 | 100 | 100 |

**Disclaimer/Publisher's Note:** The statements, opinions and data contained in all publications are solely those of the individual author(s) and contributor(s) and not of MDPI and/or the editor(s). MDPI and/or the editor(s) disclaim responsibility for any injury to people or property resulting from any ideas, methods, instructions or products referred to in the content.
